# The Impact of Lower Back Pain on Hip Extension and Lumbosacral Lateral Flexion in Junior Gymnasts during Back-Bending

**DOI:** 10.5114/jhk/196318

**Published:** 2025-07-21

**Authors:** Kazuaki Kinoshita, Yuichi Hoshino, Naoko Yokota, Masayuki Fukuda, Mika Hirata, Yuichiro Nishizawa

**Affiliations:** 1 Department of Physical Therapy, Faculty of Rehabilitation, Shijonawate Gakuen University, Osaka, Japan.; 2 Department of Orthopaedic Surgery, Graduate School of Medicine, Kobe University, Hyogo, Japan.; 3 Department of Rehabilitation, Kishimoto Orthopedic Rehabilitation Clinic, Osaka, Japan.; 4 Center of Rehabilitation, Kobe Kaisei Hospital, Hyogo, Japan.; 5 Department of Orthopaedic Surgery, Hiro Sports Clinic, Hyogo, Japan.

**Keywords:** injury, overuse, spine, range of motion, hyperextension

## Abstract

We sought to compare the spine and lower limb alignment using inertial measurement units (IMUs) in athletes with and without low back pain (LBP). Fifty junior gymnasts were divided into two groups: those with (n = 12) and without LBP (n = 38). IMU sensors were placed throughout the body. Participants were instructed to perform back-bending. The movements of shoulder, thoracolumbar, lumbosacral, hip, and knee joints were assessed. Additionally, differences between thoracolumbar and lumbosacral joints were evaluated. In the sagittal plane, lumbosacral extension was greater in the no-LBP group (37.7° ± 13.6°) than in the LBP group (24.6° ± 20.4°, p < 0.05, d = 0.85). Thoracolumbar extension was similar in the no-LBP (74.1° ± 14.4°) and LBP groups (84.5° ± 20.4°, p > 0.05, d = 0.66). The difference between thoracolumbar and lumbosacral extension was 36.4° ± 22.4° in the no-LBP group and 59.8° ± 34.2° in the LBP group, which was statistically significant (p < 0.05, d = 0.41). Hip extension was greater in the no-LBP group (10.7° ± 7.1°) than the LBP group (5.5° ± 7.6°, p < 0.05, d = 0.73). In the frontal plane, diminished lumbosacral joint lateral flexion was observed in the no-LBP group (5.8° ± 4.6°) compared to the LBP group (11.1° ± 8.3°, p < 0.05, d = 0.45). Junior gymnasts with LBP demonstrated reduced ranges of motion in hip and lumbosacral extension, along with further extension of the thoracolumbar beyond the lumbosacral joints while back-bending. Additionally, lateral flexion was observed at lumbosacral joints.

## Introduction

The prevalence of low back pain (LBP) in gymnasts is quite high, ranging 72–82.3% (Fari et al., 2021; Sabeti et al., 2015). While ankle injuries occur most frequently, the low back is most frequently painful (Andreu et al., 2022; Fari et al., 2021; Sabeti et al., 2015). One significant reason for the elevated prevalence of LBP is participation in gymnastic exercises that involve repeated joint hyperextension (Bono, 2004; Curtis and D’Hemecourt, 2007). Further aspects of LBP in gymnasts warrant consideration. Researchers have observed that many gymnasts with LBP continue to participate in their sport without altering their routine training despite pain (Harringe et al., 2004). Ignoring LBP can lead to training losses, ultimately impacting competitive performance. In professional sports, LBP is the most common cause of lost playing time (Bono, 2004; Bernstein and Cozen, 2007).

Bruggemann's radiological study (2010) showed that female gymnasts aged 12–13 years reported the most spinal abnormalities. One-third of them had severe and another third had moderate spinal abnormalities (Bruggemann, 2010). The reason for such a high prevalence of spinal abnormalities at a young age may be due to ”early” participation in competitive contexts (Baxter-Jones et al., 2003; Malina et al., 2013). Ciullo and Jackson (1985) suggested that repetitive hyperextension of the spine and associated microtrauma might cause spinal injuries. In order to ensure the safety of athletes and to minimize the risk of injuries, identifying how they should perform back-bending safely is essential.

An inertia measurement unit (IMU)-based system is preferable for analyzing low back pain (LBP) in gymnasts. An IMU-based system has emerged as a prominent tool for collecting kinematic data of multiple joints in various activities without being restricted to the laboratory environment (Feletti et al., 2023; Horsley et al., 2021; Ziwei et al., 2022). IMU devices are lightweight and can be worn by gymnasts without restricting their movement. Additionally, IMUs can be used on various surfaces and apparatuses, providing comprehensive data for different gymnastic activities (Campbell et al., 2023). For analyzing LBP in gymnasts, IMUs capture variables such as spinal extension angles, hip and knee joint movements, and the synchronization of these movements during performance. These data allow for a detailed comparison of movement patterns between gymnasts with and without LBP. The validity and reliability of IMUs in capturing these kinematic variables are well-documented, demonstrating their effectiveness for field assessments and providing accurate insights into the biomechanical alignment associated with LBP in athletes (Roetenberg et al., 2013; Vermeulen et al., 2023). The objective of this study was to compare the spine and lower limb alignment using IMUs in athletes with and without LBP. Specifically, we examined characteristics of the bent-back position alignment in junior gymnasts who experienced extension-related LBP within the previous three months. Our hypothesis was that athletes experiencing LBP would have greater spinal extensions and stiffer movements of hip and knee joints.

## Methods

Fifty junior gymnasts (12 males and 38 females) were included in the study. The average age was 12.1 ± 1.4 years, the average body height was 139.3 ± 8.1 cm, the average body mass was 32.9 ± 6.3 kg, and the average training experience was 6.6 ± 2.5 years. The LBP group consisted of 12 gymnasts, while the no-LBP group included 38 participants (Table 1). Participants trained approximately 18–25 hours per week. The demographic characteristics of the groups did not differ significantly (Table 1).

**Table 1:** Characteristics of participants.

		Total			No-LBP group			LBP group		
Age	(year)	12.1	±	1.4	12.1	±	1.4	12.0	±	1.3
Body height	(cm)	139.3	±	8.1	139.7	±	0.1	137.8	±	0.1
Body mass	(kg)	32.9	±	6.3	33.1	±	6.5	32.3	±	6.2
Training experience	(year)	6.6	±	2.5	6.4	±	2.3	7.2	±	3.2

Data are reported as mean ± SD unless otherwise indicated

This study was approved by the ethics committee of the Shijonawate Gakuen University (approval number: 22-6; approval date: 13 September 2022). It adhered to the ethical standards of institutional and national research committees as well as the 1964 Helsinki Declaration and its later amendments. Informed consent was obtained from parents, guardians, and children participating in the study.

Participants were recruited from three local gymnastic clubs in Hyogo, Japan, with competitive levels ranging from regional tournaments to national championships. Exclusion criteria included a history of spinal surgery or lower limb pathology to ensure adherence to the study protocol. Individuals with undiagnosed pain conditions or those currently experiencing LBP that caused time-loss injuries (defined as missing a scheduled session) were also excluded. Inquiries were made to determine whether participants had experienced extension-related LBP in the past 12 months. Lower back pain was defined as pain lasting more than one week. Participants were then divided into two groups for comparative analysis: those with recent extension-related LBP over the past three months (LBP group) and those without LBP for the past 12 months (no-LBP group)

### Apparatus

**Figure 1:**
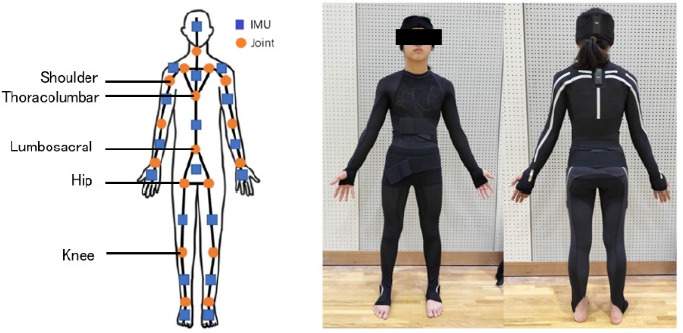
The e-skin MEVA was equipped with 18 inertial measurement unit (IMU) sensors embedded in the garment, allowing for initiation of measurements within 30 s of wearing it. The shoulder, thoracolumbar, lumbosacral, hip, and knee joints were studied. The angle of each joint was computed based on data acquired from segments equipped with IMUs positioned on both sides. This IMU system demonstrated a measurement accuracy of < 2.40° for the root mean square error and < 1.60° for the mean range of motion error in lower limb joint kinematics. Additionally, this algorithm for the calculation of three-dimensional joint angles based on gyroscope and accelerometer data from mounted IMUs showed good to excellent agreement when compared to a common optical motion capture system.

Eighteen IMU sensors (e-skin MEVA; Xenoma Inc., Tokyo, Japan) installed in e-textile segments were secured in hats, shirts, and pants fitted to participants as shown in Figure 1. Each IMU sensor contained a triaxial accelerometer and a triaxial gyroscope, enabling the estimation of three-dimensional joint kinematics and global positioning according to a known algorithm (Teuf et al., 2018). To calibrate a three-dimensional model calculation prior to measurement, each participant was asked to adopt three postures: leaning forward with their hands pressed against a wall, a bowing posture, and standing upright. The measurement datasets comprised raw IMU sensor signals (acceleration and angular velocity), global positioning of each sensor and anatomical landmarks, as well as joint angles involving the ankle, knee, hip, lumbosacral, thoracolumbar, sternoclavicular, neck, wrist, elbow, and shoulder joints. The definitions of movements were as follows: in the sagittal plane, '+' denoted flexion and '-' denoted extension; in the frontal plane, '+' indicated abduction and '-' indicated adduction; and in the horizontal plane, '+' represented external rotation and '-' represented internal rotation. However, thoracolumbar and lumbosacral movements were indicated by '+,' as they were specifically defined as lateral bending in the frontal plane.

### Procedures

Measurements of joint angles were performed when participants adopted the bent-back position. Participants were instructed to hold both upper and lower limbs as close as possible and to straighten the body. Participants practiced several times before measurements until reaching a full understanding of the required posture.

### Data Collection

Data were collected for 5 s, and the average values during the intermediate 3 s were used for further analysis. The joint angles of the shoulder, the thoracolumbar, the lumbosacral, the hip, and the knee were analyzed (Figure 1). Additionally, to explore the hypothesis of excessive lumbar curvature, a variable measuring the difference between the thoracolumbar and lumbosacral (Th-L) joints was introduced. Since all participants exhibited lateral bending, measurements of joint angles at the shoulder, the hip, and the knee were analyzed on both the bent and opposite sides. The internal consistency of the IMU measurements was previously assessed, showing intraclass correlation coefficients (ICCs) ranging from 0.52 to 0.99 for all joint angles measured, indicating high repeatability (Teufl et al., 2018). The IMU measured joint angles for the shoulder, the thoracolumbar, the lumbosacral, the hip, and the knee, expressed in degrees. The measurement error of the IMU was generally less than 1°, thus the values were reported to one decimal place (Teufl et al., 2018).

### Statistical Analysis

Measurements were analyzed with descriptive statistics using mean values and standard deviations. The ICCs were calculated to assess intra-rater repeatability of the bent-back position. Values were analyzed using one-way analysis of variance (ANOVA) with 95% confidence intervals. ICCs were interpreted according to Koo and Li (2016) where ICC > 0.90 = excellent, 0.75–0.90 = good, 0.50–0.74 = moderate and < 0.50 = poor. Table 2 presents the ICCs for this study. ICCs for the anterior-posterior and horizontal planes of the shoulder and knee joints were 0.74 or below. Consequently, these variables were omitted from further analysis.

**Table 2:** Reproducibility of joint positions in the back-bend position.

		ICC (1.1)	95%CI	
Lower	Upper
Shoulder	sagittal	0.98	0.87	1.00
	frontal	0.47	−0.06	1.00
	horizontal	0.63	0.05	1.00
Thoracolumbar	sagittal	0.78	0.23	1.00
	frontal	0.88	0.43	1.00
	horizontal	0.75	0.18	1.00
Lumbosacral	sagittal	0.90	0.51	1.00
	frontal	0.88	0.44	1.00
	horizontal	0.97	0.82	1.00
Hip	sagittal	0.91	0.52	1.00
	frontal	0.75	0.18	1.00
	horizontal	0.95	0.70	1.00
Knee	sagittal	0.95	0.70	1.00
	frontal	0.55	−0.01	1.00
	horizontal	0.54	−0.02	1.00

Statistical analysis was conducted using IBM SPSS Statistic 20 (IBM, New York, USA). Before analyzing the differences, normality of data distribution was tested using the Shapiro-Wilk test. To determine differences between LBP and no-LBP groups, the Student’s *t*-test was used for independent samples for normally distributed variables and the Mann-Whitney U-test for non-normally distributed variables. The level of significance was set at α < 0.05. Statistical power analysis was conducted using G*Power software (version 3.1.9.2) with a significance level (α) of 0.05. Considering that the sample sizes were 12 in the LBP group and 38 in the no-LBP group, assuming a medium effect size of 0.8, the calculated sample power was 0.66%.

## Results

In the sagittal plane, the lumbosacral extension was 37.7° ± 13.6° in the no-LBP group and 24.6° ± 20.4° in the LBP group, with a statistically significant difference (*p* < 0.05, *d* = 0.85) (Table 3). The effect size (*d* = 0.85) indicated a large effect, suggesting that the difference in lumbosacral extension between the groups was substantial. No significant difference was observed in thoracolumbar extension (74.1° ± 14.4° in the no-LBP group and 84.5° ± 20.4° in the LBP group, *d* = 0.66). However, the difference between thoracolumbar and lumbosacral extension was lower in the no-LBP group (36.4° ± 22.4°) compared to the LBP group (59.8° ± 34.2°, *p* < 0.05, *d* = 0.41) (Table 4). Hip extension was greater in the no-LBP group (10.7° ± 7.1°) than the LBP group (5.5° ± 7.6°, *p* < 0.05, *d* = 0.73) (Table 5). The effect size (*d* = 0.73) represented a large effect, highlighting the significant difference in hip extension between the two groups. In the frontal plane, lateral flexion of the lumbosacral was 5.8° ± 4.6° in the no-LBP group and 11.1° ± 8.3° in the LBP group, revealing a statistically significant difference (*p* < 0.05, *d* = 0.45) (Table 3). Decreased hip extension was compensated by increased thoracolumbar extension.

**Table 3:** Comparison of thoracolumbar and lumbosacral angles.

		No-LBP group				LBP group				Effect Size	95%CI		*p*
Lower	Upper
Thoracolumbar	sagittal	−74.1	±	14.4		−84.5	±	20.4	0.66	−0.03	1.34	n.s.
	frontal	6.6	±	5.0		11.3	±	7.7	0.39	−0.66	−0.02	n.s.
	horizontal	6.0	±	5.9		6.5	±	5.2	0.12	−0.47	0.27	n.s.
Lumbosacral	sagittal	−37.7	±	13.6		−24.6	±	20.4	0.85	−1.54	−0.16	*p* < 0.05
	frontal	5.8	±	4.6		11.1	±	8.3	0.45	−0.70	−0.09	*p* < 0.05
	horizontal	4.3	±	4.1		4.1	±	3.8	0.10	−0.45	0.29	n.s.

Angle definition: sagittal plane, flexion '+' and extension '-'; frontal plane, lateral flexion '+'; horizontal plane, rotation to the side with lateral flexion '+'

**Table 4:** Comparison of the difference between thoracolumbar and lumbosacral angles.

			No-LBP group				LBP group				Effect Size	95%CI		*p*
Lower	Upper
Th-L	sagittal	−36.4	±	22.4		−59.8	±	34.2	0.41	0.05	0.68	*p* < 0.05
	frontal	0.8	±	5.3		0.2	±	6.6	0.10	−0.57	0.77	n.s.
	horizontal	1.6	±	7.5		2.4	±	7.3	0.11	−0.78	0.56	n.s.

Th-L: difference between the thoracolumbar and the lumbosacral

**Table 5:** Comparison of hip, knee and shoulder angles.

			No-LBP group				LBP group			Effect Size	95%CI		*p*
											Lower	Upper	
lateral flexion side	Shoulder	sagittal	127.8	±	10.0		133.1	±	11.6	0.52	−1.22	0.19	n.s.
	Hip	sagittal	−10.7	±	7.1		−5.5	±	7.6	0.73	−1.41	−0.04	*p* < 0.05
		frontal	7.9	±	10.6		11.2	±	11.1	0.31	−1.09	0.46	n.s.
		horizontal	2.1	±	10.5		4.2	±	9.1	0.21	−0.91	0.49	n.s.
	Knee	sagittal	74.0	±	9.9		74.8	±	9.8	0.09	−0.79	0.62	n.s.
no lateral flexion side	Shoulder	sagittal	129.5	±	11.6		125.2	±	8.7	0.39	−0.32	1.09	n.s.
	Hip	sagittal	−10.5	±	5.9		−7.3	±	7.0	0.51	−1.18	0.17	n.s.
		frontal	8.4	±	8.8		3.0	±	5.3	0.65	−0.10	1.39	n.s.
		horizontal	3.6	±	7.8		−1.1	±	6.6	0.61	−0.08	1.30	n.s.
	Knee	sagittal	74.4	±	13.0		76.7	±	9.7	0.18	−0.88	0.53	n.s.

Lateral flexion side: Indicates lateral flexion on the frontal plane in the thoracolumbar; Angle definition: sagittal plane, flexion '+' and extension '-'; frontal plane, abduction '+' and adduction '-'; horizontal plane, '+' external rotation and '-' internal rotation

## Discussion

Junior gymnasts with LBP demonstrated reduced hip and lumbosacral extension when performing a bent-back position, along with further thoracolumbar extension beyond the lumbosacral joint. Additionally, lateral flexion was noted at the lumbosacral joint. This could lead to LBP if the bent-back position exhibited inadequate extension angles in specific regions or if the trunk underwent lateral flexion. The novelty of this study, in particular, lies in the observation of lateral flexion in the back-bending position among individuals experiencing LBP. These results do not fully support the hypothesis that athletes with LBP would show excessive spinal extension and stiffer hip and knee joint movements. However, they do suggest a distinct movement pattern in the bent- back position.

The bent-back position with insufficient hip extension may impose mechanical stress on the thoracolumbar joint, resulting in LBP. This rationale lies in the fact that the bent-back position, with selective extension of certain joints, results in localized stress within those specific areas. Ciullo and Jackson (1985) postulated that repetitive hyperextension and microtrauma of the spine were potential contributors to spinal injuries. A two-dimensional kinematic study of national team female gymnasts revealed that those with LBP exhibited slightly greater mobility involving the lumbar spine, combined with less flexibility in the thoracic and hip extension (Brady and Vicenzino, 2002). Furthermore, Sands et al. (2016) reported that the inclusion of specialized stretching to improve thoracic and hip extension may reduce the risk of lower back pain in gymnasts, rather than contribute to it. These studies support our research confirming that bent-back position geometry causes lumbar problems.

Hyperextension of the trunk does not necessarily cause lumbar injuries. Tertti et al. (1990) identified only three instances of observable disc regression in an magnetic resonance imaging (MRI) study involving 35 prepubertal gymnasts. Those authors concluded that despite the presence of an excessive range of motion and substantial axial loading of the spine, primary damage to intervertebral discs was infrequent in young gymnasts (Tertti et al., 1990). Bennett et al. (2006) reported similar findings in Olympic-level gymnasts aged 12–20 years. A study involving rhythmic gymnasts aged 13–19 years revealed that factors such as youth, greater leanness, nonsmoking status, reduced anxiety and depressive behavior, as well as enhanced muscle strength and flexibility were all associated with a lower incidence of LBP. The findings from that study suggest that rhythmic gymnastics did not pose an increased risk of LBP (Cupisti et al., 2004). Contrary to conventional wisdom, retired rhythmic gymnasts do not exhibit a higher incidence of LBP when compared with age-matched controls (Piazza et al., 2009). In other words, numerous cases of sports involving hyperextension without resulting in lumbar injuries have been reported. These observations indicate that back bending would not harm the lower back if it is performed in an appropriate fashion.

One of the most striking findings of this study was the presence of lateral flexion in the bent-back position within the LBP group. Most prior reports had exclusively focused on the sagittal plane. The spine is a three-dimensional structure, which bends dynamically with natural curves in the sagittal (flexion/extension), frontal (lateral flexion), and horizontal (twisting left or right) planes. Acknowledging the significance of evaluating spinal motion not solely in the sagittal plane, but also in the frontal and horizontal planes is important. Previous research has included radiographic and MRI studies (Tertti et al., 1990), kyphometer and inclinometer studies (Ohlen et al., 1989), and studies using optical raster-stereographic methods (Wojtys et al., 2000). Therefore, exploring spinal movements in the frontal and horizontal planes at the same time is challenging. Our motion analysis using IMUs examined whole-body movement multidimensionally using a suit, providing novel findings. Lumbosacral lateral flexion is assumed to impose uneven stress on specific intervertebral joints. In recent years, there has been a critical discourse surrounding the adverse relationship between postural distortions and pain. Only a small fraction of individuals with postural distortions experiences LBP. A study of the sitting posture reported no relationship between the pelvic tilt and pain (Tae-sung et al., 2021). However, the bent-back position represents a bridge activity wherein the back muscles are activated more prominently. The back muscles often cause myofascial back pain. Uneven force exertion on back muscles can lead to overuse and subsequent pain on one side.

This study has certain limitations that should be acknowledged. First, the methodology of this study exhibited good reproducibility; however, there were discrepancies in terms of values of the results compared to kinematic numerical data. This discrepancy is attributed to the method of segment assembly (Figure 1). Considering the inherent differences in meaning between the actual kinematics and the measured values is important. One methodological issue in this paper pertains to the poor reproducibility of movements in the frontal and horizontal planes for the shoulder and knee joints. The suboptimal ICC values may be attributed to characteristics of the attire worn during measurements. While suits exhibit minimal shifting along the longitudinal axis, potential deviations are anticipated due to the flexibility of certain areas and the slender physique of participants, leading to garment distortion. For these reasons, the frontal and horizontal movements of the shoulder and the knee were excluded from the variables considered in this study. This exclusion ensured the validity of the methodology employed in this research. Second, as this was a cross-sectional study, causation cannot be definitively established involving the examined factors. Consequently, firm conclusions cannot be drawn at present. Future investigations should proactively explore whether distortions in the basic posture of gymnasts, particularly the posterior bridge position, are associated with the onset of LBP. Third, the history of prior LBP relies on participants' self-reported responses, potentially introducing recall bias, as participants may not accurately remember events occurring up to one year ago. Additionally, strict consideration of spinal alignment abnormalities (e.g., scoliosis) among the study participants was lacking. Notably, participants, being gymnasts engaged in regular training, were selected for their apparent absence of gross orthopedic abnormalities. Therefore, significant spinal alignment abnormalities are presumed to be absent in this cohort. Moreover, contemporary gymnasts often emphasize rapid extension and flexion movements and performance during competition is predominantly characterized by dynamic variations. Whether static and dynamic postures, such as those investigated in this study, are entirely linked remains uncertain. While we believe that the imposition of a complete bridge position is relevant to gymnastic activities such as somersaults, which involve dynamic movements, future research should also consider the movements of individual joints during dynamic postures. Lastly, gymnastics involves the execution of high-force movements combined with unstable landing positions or falls. The injury patterns in gymnasts cannot be conclusively attributed solely to spinal flexibility. This study only offers insights into LBP arising from various factors and should not be considered as a conclusive examination of a single posture. Finally, although the effect observed in this study was statistically significant, the small sample size may have limited the ability to detect larger effects. Additionally, the large standard deviations suggest that there may be room for improvement in the study design and evaluation methods.

## Conclusions

Junior gymnasts exhibiting a bent-back position with LBP demonstrated a reduced range of motion in the hip and lumbosacral extension, along with further extension of the thoracolumbar joint beyond the lumbosacral spine. Additionally, lateral flexion was noted at the lumbosacral joints. The findings of this study enhance our understanding of LBP in sports and lay the groundwork for future research. They may inform strategies for preventing and managing LBP in gymnasts, particularly by optimizing the spinal posture and movement. However, the effectiveness of these strategies should be confirmed through further research. Future studies should examine the relationship between changes in the gymnasts’ posture and the onset of LBP in more detail, considering dynamic movements and performance during competitions. Utilizing larger sample sizes and a broader range of variables will contribute to a more comprehensive understanding of LBP in gymnasts.
